# Evolutionary History of *Helicobacter pylori* Sequences Reflect Past Human Migrations in Southeast Asia

**DOI:** 10.1371/journal.pone.0022058

**Published:** 2011-07-19

**Authors:** Sebastien Breurec, Bertrand Guillard, Sopheak Hem, Sylvain Brisse, Fatou Bintou Dieye, Michel Huerre, Chakravuth Oung, Josette Raymond, Tek Sreng Tan, Jean-Michel Thiberge, Sirenda Vong, Didier Monchy, Bodo Linz

**Affiliations:** 1 Unité de Biologie Médicale et Environnementale, Institut Pasteur, Dakar, Senegal; 2 Unité de Biologie Médicale, Institut Pasteur, Phnom Penh, Cambodia; 3 Plate-Forme Génotypage des Pathogènes et Santé Publique, Institut Pasteur, Paris, France; 4 Unité de Recherche et d'Expertises en Histotechnologie et Pathologie, Institut Pasteur, Paris, France; 5 Gastroenterology and Liver Unit, Calmette Hospital, Phnom Penh, Cambodia; 6 Unité Postulante Pathogenèse de Helicobacter, Institut Pasteur, Paris, France; 7 Université Paris Descartes, Faculté de Médecine, Paris, France; 8 Private Medical Center, Phnom Penh, Cambodia; 9 Unité d'Epidémiologie et de Santé Publique, Institut Pasteur, Phnom Penh, Cambodia; 10 Laboratoire de Biologie Médicale, Institut Pasteur, Bangui, République Centrafricaine; 11 Department of Biochemistry and Molecular Biology, Pennsylvania State University, University Park, Pennsylvania, United States of America; University of Edinburgh, United Kingdom

## Abstract

The human population history in Southeast Asia was shaped by numerous migrations and population expansions. Their reconstruction based on archaeological, linguistic or human genetic data is often hampered by the limited number of informative polymorphisms in classical human genetic markers, such as the hypervariable regions of the mitochondrial DNA. Here, we analyse housekeeping gene sequences of the human stomach bacterium *Helicobacter pylori* from various countries in Southeast Asia and we provide evidence that *H. pylori* accompanied at least three ancient human migrations into this area: i) a migration from India introducing hpEurope bacteria into Thailand, Cambodia and Malaysia; ii) a migration of the ancestors of Austro-Asiatic speaking people into Vietnam and Cambodia carrying hspEAsia bacteria; and iii) a migration of the ancestors of the Thai people from Southern China into Thailand carrying *H. pylori* of population hpAsia2. Moreover, the *H. pylori* sequences reflect iv) the migrations of Chinese to Thailand and Malaysia within the last 200 years spreading hspEasia strains, and v) migrations of Indians to Malaysia within the last 200 years distributing both hpAsia2 and hpEurope bacteria. The distribution of the bacterial populations seems to strongly influence the incidence of gastric cancer as countries with predominantly hspEAsia isolates exhibit a high incidence of gastric cancer while the incidence is low in countries with a high proportion of hpAsia2 or hpEurope strains. In the future, the host range expansion of hpEurope strains among Asian populations, combined with human motility, may have a significant impact on gastric cancer incidence in Asia.

## Introduction

The fragmented distribution of speakers of the five major language families in Southeast Asia is the result of extensive human migrations. Hmong Mien, Austro-Asiatic and Austronesian are considered the older language families in the region [1], whereas the presence of the Sino-Tibetan and Tai-Kadai language families can be attributed to relatively recent population expansions. Most fragmented is the distribution of Hmong-Mien speakers living in numerous small enclaves surrounded by Sino-Tibetan and Tai-Kadai speakers in Southern China, Laos and Northern Vietnam because of an extreme expansion of the Chinese subfamily of Sino-Tibetan (mostly during the Zhou dynasty 1100 to 221 BC) which distributed Chinese languages continuously over a large region from North to South China, pushing speakers of other languages further south and west. The Austro-Asiatic language family (with the examples of Vietnamese from Vietnam and Khmer from Cambodia) was previously distributed from Vietnam in the east and South China in the north to the Malay Peninsula in the south and North India to the west [2] before massive expansions of Indo-European speakers in India and Tibeto-Burman speakers (a subgroup of Sino-Tibetan different from Chinese) from South China into Myanmar restricted Austro-Asiatic languages to numerous enclaves in this area. A subsequent expansion of Tai-Kadai speakers during the early second millennium AD from their homeland in South China into Thailand and Laos replaced Austro-Asiatic speakers in large parts of Southeast Asia that previously belonged to the Khmer empire [3], [4], [5]. Subsequently, Tai-Kadai is found from South China over Thailand to the Malay Peninsula and Myanmar.

In historic times, parts of Southeast Asia have repeatedly been ruled by colonial forces, but there has never been overall occupation [1], [4]. The Han Chinese invaded North Vietnam (Tonkin) in the 1^st^ century BC and stayed for nearly a millennium, after which Vietnamese dynasties from North Vietnam conquered central Vietnam (Annam) and South Vietnam (Cochin China). The French occupied the same area (Tonkin, Annam, Cochin China) during a far shorter period (1863–1953), and added present day Cambodia and Laos to their colonial French Indochina. Both of these colonial episodes excluded Siam (Thailand), the only country in Southeast Asia never colonized by a European power.

Archaeology suggests an ancient close connection between India and the Thailand/Cambodia region through settlement [6], [7], [8], [9], accompanied by an increasing exposure to Indian culture from about 300 BC. Early states-like societies from Southeast Asia called by the Sanskrit term “mandala” had in common the adoption of Indian forms of religion (Hinduism), the Sanskrit language and aspects of government (Funan mandala from 100 to 550 AD, Chenla mandala from 550 to 802 AD and Angkorian mandala from 802 to 1431 AD) [4]. However, the Indian influence in Southeast Asia was not supported by human mitochondrial DNA (mtDNA) data [10], [11], [12].

In previous studies, we have used housekeeping gene sequences of a bacterial parasite which infects the stomach of most humans, *Helicobacter pylori*, to elucidate the patterns of human prehistory. *H. pylori* accompanied modern humans during their migrations out of Africa ca. 60,000 years ago [13], and subsequent geographic separation plus founder effects have resulted in genetic populations of bacterial strains that are specific for large continental areas. In all, 7 bacterial genetic populations have been described [13], [14], [15], [16], [17], [18]: hpEurope (isolated from Europe, the Middle East, India and Iran), hpNEAfrica (isolated in Northeast Africa), hpAfrica1 (isolated from countries in Western Africa and South Africa), hpAfrica2 (so far only isolated from South Africa), hpAsia2 (isolated from Northern India and among isolates from Bangladesh, Thailand and Malaysia), hpSahul (from Australian Aboriginals and Papua New Guineans) and hpEastAsia with the subpopulations hspEAsia (from East Asians), hspMaori (from Taiwanese Aboriginals, Melanesians and Polynesians) and hspAmerind (Native Americans). All these modern populations derived from six ancestral populations that were designated ancestral Europe1 (AE1), ancestral Europe2 (AE2), ancestral EastAsia, ancestral Africa1, ancestral Africa2 [14] and ancestral Sahul [16].

The specific geographic distribution and ethnic association of the *H. pylori* populations reflects numerous ancient and historic human migrations which established *H. pylori* sequences as a useful genetic marker to unravel debated topics in human population history. For example, the genetic variation in *H. pylori* has showed more discriminatory power in determining the ancient sources of human migrations in the Ladakh region of Northern India [19] and in the Pacific (Austronesian expansion) [16] than traditional human genetic markers such as the hypervariable region (HSV1) of mtDNA. Therefore, we analysed *H. pylori* sequences from Cambodia which borders Thailand to its west and northwest, Vietnam to its east and southeast and Laos to its north, to gain additional insights into the human population history in continental Southeast Asia.

## Materials and Methods

### Strains and ethics statement

Gastroduodenal endoscopy was performed at the Gastroenterology Department of the Calmette Hospital and at a private medical center in Phnom Penh, Cambodia with the permission of the Cambodian National Ethics Committee for Health Research (ethics certificate 017/03NECHR). Informed written consent was received from all participants.

A total of 66 *H. pylori* strains derived from 66 patients (36 (55%) males, median age 46.0 years; range 18–76 years) who suffered from upper abdominal pain were isolated in 2004 and in 2007. Demographic data, the medical history and the presenting symptoms were prospectively collected by the physician. All the patients were of Khmer origin, and none had received proton pump inhibitors or antibiotics during the 4 weeks before endoscopy. Three biopsy samples were taken from the antrum and three from the fundus during upper gastrointestinal tract endoscopy. One biopsy from each site was cultured for *H. pylori* isolation, and the others were fixed and processed for histological analysis.

The Cambodian strains were supplemented by unpublished sequences of strains from French Caucasians (n = 8), as well as sequences obtained from http://pubmlst.org/helicobacter/ that were previously published by Falush *et al.* 2003 [14], Wirth *et al.* 2004 [15], Momynaliev *et al.* 2005 [20], Linz *et al.* 2007 [13], Devi *et al.* 2007 [17], Tay *et al.* 2009 [18], Liao *et al.* 2009 [21] and Moodley *et al.* 2009 [10]. Novel sequences experimentally obtained in this study were deposited in GenBank database under the following accession numbers HM362684 to HM362767.

### *H. pylori* isolates and genomic DNA

*H. pylori* culture was performed using Columbia agar plates with 10% (v/v) defibrinated horse blood and *H. pylori* selective antibiotic supplement (Oxoid, Basingstoke, UK) containing vancomycin (10 mg/L), cefsulodin (5 mg/L), trimethoprim (5 mg/L) and amphotericin B (5 mg/L). The plates were incubated for up to 10 days at 37°C under microaerophilic conditions (GENbag, Biomerieux). *H. pylori* was identified by colony and microscopic morphology and by positive urease, catalase, and oxidase tests. From primary growths, a single *H. pylori* colony from antrum or fundus was picked and subcultured in order to ensure that each strain consists of only a single genotype. Genomic DNA was extracted using a QIAmp™ kit (Qiagen, Courtaboeuf, France).

### Data analysis

PCR amplification and sequencing of *atpA*, *efp*, *mutY*, *ppa*, *trpC*, *ureI*, and *yphC* were performed as previously described [13]. Strain population assignment was performed as described by Falush et al [14] using the “no admixture model” of Structure [22]. The linkage model in Structure was used to estimate the proportion of nucleotides being derived from each ancestral population as described [13], [14]. The estimated amount of ancestry from each population was plotted as a thin line for each isolate using Distruct [23].

Pair-wise *F_ST_* values as well as the analyses of molecular variance (AMOVA) were calculated in Arlequin [24] as described before [25] using the Kimura 2-parameter model that was previously applied to *H. pylori* sequences [13], [14], [19], [25]. The significance of the pair-wise *F_ST_* values was estimated by running 10,000 permutations assuming no difference between the populations. Neighbor-joining trees from the pair-wise *F_ST_* values were generated in Mega v4 [26].

## Results and Discussion

### *H. pylori* from Khmer in Cambodia

*H. pylori* isolates were cultured from gastric biopsies obtained from 66 Khmer volunteers during gastroduodenal endoscopy at the Calmette Hospital (n = 37) and at a private medical center (n = 29) in Phnom Penh, Cambodia, in 2004 and 2007. The concatenated sequences of 7 housekeeping gene fragments (3406 base pairs, of which 838 were polymorphic) yielded 66 unique haplotypes that were compared to haplotypes from other countries in Asia and ∼700 haplotypes from other sources including Europe and Sahul. Bayesian clustering algorithms implemented in Structure (no admixture model) [18] assigned 34 (52%) new bacterial haplotypes to the *H. pylori* population hpEurope and 32 (48%) new haplotypes to hpEastAsia, subpopulation hspEAsia (Table 1), with no significant difference between 2004 and 2007 (data not shown). The large proportion of hpEurope strains is surprising because *H. pylori* from this population are known to be more characteristic of the Middle East, Europe and countries colonized by Europeans [13], [14], India [17] and central Asia including Iran [25]. Given the large geographical origin of the patients attending the study health facility, we believe that the sample may be representative of the country.

**Table 1:** Sources and population assignment of the analysed *H. pylori* strains.

	*Number of isolates assigned to*					
Country/ethnic origin	hpEurope	hpAsia2	hspEAsia	hspMaori	hspAmerind	Reference
Iran	125					27
Kazakhstan	6					13
Turkey	18					13
Estonia	10					14
Finland	10					14
Germany	23					14
Netherlands	5					13
UK	16					14
Russia	21					20
France	8					this study
Italy	9					13
Spain Spaniard	35					14
Spain Basque	44					13
Lebanon	5					13
Palestine	11					13
Thailand Thai	6	9				13
Thailand Chinese	1		18			13
India (Ladakh) Muslim	2	14				19
India (Ladakh) Buddhist		25	6			19
India (Andhra Pradesh)	23					17
Malaysia Indian	8	23	4			18
Malaysia Malay	4	9	2			18
Malaysia Chinese	1		25			18
Singapore Chinese			9			13
Taiwan Chinese			15			13
Cambodia	34		32			this study
Philippines	7	3				13
Bangladesh		3				13
Vietnam	2		20			13
Japan			24			13
China	5		93			21, 13
Korea			10			14
Taiwan Aboriginal				43		16
New Caledonia*				6		16
Wallis Polynesians				4		16
Samoa Polynesians				8		14
New Zealand Maori				15		14
North America					11	13
South America					7	13, 42

*Melanesians.

**Figure 1:**
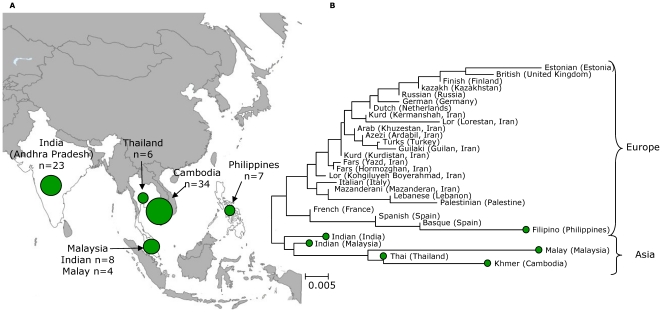
The distribution and phylogenetic differentiation of European (hpEurope) *H. pylori* isolates in Southeast Asia. (A) Map of sampling locations of hpEurope haplotypes in Southeast Asia. (B) The Neighbor-joining tree generated from pair-wise *F_ST_* values indicates a common, non-European origin of the Southeast Asian hpEurope strains.

**Figure 2:**
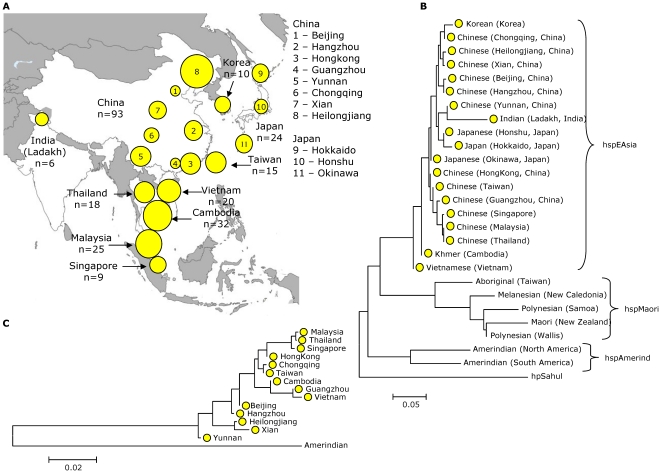
hpEastAsia *H. pylori* strains in Southeast Asia. (A) Map of sampling locations of hspEAsia haplotypes in Southeast Asia. The size of the chart indicates the number of hspEAsia haplotypes at each sampling location. (B) Neighbor-joining tree from pair-wise *F_ST_* values of hpEastAsia haplotypes rooted with haplotypes of the population hpSahul. (C) Neighbor-joining tree of pair-wise *F_ST_* values of the subpopulation hspEAsia.

Strains of the hpEurope population were shown to be hybrids of two ancestral populations, AE1 from central Asia and AE2 from northeast Africa [13] while modern hpEastAsia strains are almost pure descendants of ancestral EastAsia. By using the linkage model of Structure [22] to estimate the proportion of nucleotides derived from each of the previously identified ancestral populations [13], [14], [16], we identified isolates from Khmer that had acquired significant proportions (>20%) of foreign nucleotides from other ancestral populations. Four hspEAsia strains (12.5%) harboured a high proportion of AE2 while eight hpEurope strains (23%) contained a significant EastAsian ancestral component (Figure S1), indicating long time co-evolution of hpEurope and hspEAsia bacteria in the area. Introgressed nucleotides from other ancestral populations might change the level of differentiation between the *H. pylori* populations and thus distort the pair-wise *F_ST_* values. We stripped the dataset from isolates with a proportion of imported nucleotides from other ancestral populations >20% which did not change what populations were significantly differentiated from each other. In addition, the topology of the neighbor-joining trees (Figure 1; Figure 2) was unaffected (not shown), and there were only minor differences in the length of a few branches. Therefore, all the strains were included in the subsequent analyses.

### An ancient migration from India introduced hpEurope strains to Southeast Asia

The high prevalence of hpEurope strains (52%) in Khmer population raises the question of the origin of these isolates. If modern introduction by French during the Indochina history were the source, hpEurope strains would be expected to be widespread in Vietnam and Cambodia and scarce in Thailand, because Vietnam and Cambodia were part of the French colonial empire for a short period (1887–1954) but the kingdom of Siam (Thailand) was never under European rule. However, the frequency of hpEurope strains among ethnic Thai was higher (37%) than among Vietnamese (9%) (Table 1) [13]. In order to investigate signatures of genetic differentiation, we calculated pairwise *F_ST_* values in Arlequin [19] using concatened sequences of hpEurope strains from various countries in Europe (173 strains), from the Middle East (16 strains), from Iran (125 strains), from Cambodia (34 strains), from India (23 strains), from Malaysia (8 strains from Indians and 4 from Malays), from Thailand (6 strains from Thai) and from the Philippines (7 strains) (Figure 1A). *H. pylori* haplotypes from the Philippines, that experienced over three centuries of Spanish colonial history (1565–1898), were significantly differentiated from the Khmer and Thai populations, but not from Spanish, and thus likely resulted from a recent introduction by Europeans. In contrast, the Khmer population was not significantly differentiated from the Thai population but was significantly differentiated from European populations including French population (p<0.05) (Table 2), rejecting the hypothesis of a recent introduction of hpEurope strains by the French during the Indochina history. These observations suggest that hpEurope bacteria in Southeast Asia might be a marker for an old human migration that predated the European colonial history.

**Table 2:** Pair-wise *F_ST_* values between *H. pylori* of the population hpEurope from Europe, the Middle East and Asia.

*Country (No.)*		*1*	*2*	*3*	*4*	*5*	*6*	*7*	*8*	*9*	*10*	*11*	*12*	*13*	*14*	*15*	*16*	*17*	*18*	*19*	*20*	*21*
**Iran (125)**	**1**	-																				
**Turkey (18)**	**2**	0.003	-																			
**Estonia (10)**	**3**	**0.051**	**0.045**	-																		
**Finland (10)**	**4**	**0.043**	**0.023**	**0.013**	-																	
**Germany (23)**	**5**	**0.016**	**0.022**	**0.023**	**0.035**	-																
**Netherlands (5)**	**6**	0.002	0.003	0.030	0.028	−0.005	-															
**UK (16)**	**7**	**0.042**	**0.028**	**0.022**	0.014	**0.025**	0.020	-														
**Kazakhstan (6)**	**8**	0.013	−0.004	0.012	0.007	0.023	0.030	0.023	-													
**Russia (21)**	**9**	**0.015**	**0.025**	**0.015**	**0.030**	0.006	0.002	**0.027**	0.002	-												
**Lebanon (5)**	**10**	0.019	0.024	**0.075**	**0.061**	**0.052**	0.013	**0.047**	0.025	**0.042**	-											
**Palestine (11)**	**11**	**0.026**	**0.052**	**0.102**	**0.096**	**0.060**	**0.047**	**0.086**	**0.052**	**0.047**	−0.007	-										
**France (8)**	**12**	0.011	**0.027**	**0.032**	**0.029**	0.007	−0.015	0.022	0.012	0.002	0.025	**0.032**	-									
**Italy (9)**	**13**	**0.014**	**0.036**	**0.063**	**0.065**	0.019	0.006	**0.042**	**0.037**	**0.022**	0.017	**0.023**	0.004	-								
**Spain Spaniard (35)**	**14**	**0.026**	**0.050**	**0.067**	**0.062**	**0.026**	0.018	**0.064**	**0.043**	**0.026**	**0.035**	**0.030**	−0.003	0.015	-							
**Spain Basque (44)**	**15**	**0.040**	**0.058**	**0.074**	**0.083**	**0.040**	**0.037**	**0.075**	**0.060**	**0.041**	**0.044**	**0.041**	0.007	**0.032**	0.004	-						
**Thailand (6)**	**16**	**0.036**	**0.057**	**0.061**	**0.060**	**0.040**	0.014	**0.042**	0.031	**0.040**	**0.038**	**0.060**	−0.005	0.022	0.020	**0.045**	-					
**India (22)**	**17**	**0.031**	**0.045**	**0.057**	**0.059**	**0.026**	0.023	**0.049**	**0.039**	**0.037**	**0.044**	**0.048**	0.013	**0.026**	**0.030**	**0.049**	0.018	-				
**Malaysia Indian (8)**	**18**	**0.028**	**0.036**	**0.050**	**0.040**	0.020	0.027	**0.043**	0.024	**0.034**	0.049	**0.053**	0.011	0.030	**0.024**	**0.045**	0.006	0.013	-			
**Malaysia Malay (4)**	**19**	**0.076**	**0.067**	**0.085**	0.077	**0.079**	0.099	0.058	0.037	**0.093**	0.085	**0.128**	0.043	0.077	**0.080**	**0.088**	0.034	**0.074**	0.059	-		
**Cambodia (34)**	**20**	**0.065**	**0.076**	**0.092**	**0.079**	**0.069**	**0.066**	**0.070**	**0.077**	**0.068**	**0.075**	**0.095**	**0.035**	**0.063**	**0.060**	**0.078**	0.012	**0.049**	**0.051**	**0.061**	-	
**Philippines (7)**	**21**	**0.068**	**0.106**	**0.110**	**0.123**	**0.062**	**0.066**	**0.092**	**0.111**	**0.062**	**0.069**	**0.070**	0.030	**0.037**	0.016	0.019	**0.076**	**0.055**	**0.066**	**0.123**	**0.107**	-

Significant FSTs (significance level = 0.05) are shown in bold.

A neighbor-joining tree based on these pairwise *F_ST_* values (Figure 1B) joined the hpEurope haplotypes from the Indian, Thai, Khmer and Malay populations into a distinct cluster that was separated from haplotypes from Europe and the Middle East which indicated a common origin of these Asian hpEurope strains. Tay *et al.* [18] suggested a recent introduction of the hpEurope haplotypes by Indians into Malaysia within the last 200 years. Malaysian Indians are largely descended from people who migrated from southern India during the British colonization of Malaysia [27], and strains from modern Indians and Indians from Malaysia indeed clustered together, consistent with their origin (Figure 1B). However, strains from Malays were more closely related to those from Khmers and Thais than they were to Indian or Malaysian Indian strains, suggesting a common origin of these strains and arguing against an exclusively recent acquisition of Malaysian hpEurope strains from Indian immigrants, contrary to Tay *et al.*'s interpretation [27]. Moreover, people of Indian origin are not common in Cambodia or Thailand, a situation that contrasts with Malaysia where Indian ethnicity exceeds 7% of the general population. Strains from modern Indians and Malaysian Indians were located near the base of the branch leading to the Thai, Khmer and Malay haplotypes in the neighbor-joining tree (Figure 1B), suggesting the Indian subcontinent as the source of hpEurope bacteria in Thais, Khmers and Malays. Group assignments by AMOVA analyses for hpEurope strains provided strong statistical support of the tree topology (Table S1). Taken together, all these observations indicate an old introduction of hpEurope strains into the Indian subcontinent by Indo-Aryan migration (4000–10000 BP) as previously described [17], [28]. This was followed by subsequent eastward migrations of their descendants into Southeast Asia, carrying hpEurope strains in their stomach, probably within the last 3000 years. The hpEurope strains in Malays likely originated from both migrations, the ancient migration and a more recent migration of Indians into Malaysia.

A study on the distribution of *H. pylori* virulence factor *cagA* among Vietnamese identified 84% of the strains harbouring the type II of the cag-right motif [29] which is characteristic for East Asian strains (hpEastAsia), ranging from 76% in Ho Chi Minh city in South Vietnam to 93% in Hanoi in North Vietnam. However, there was a remarkable difference in the frequency of cag-right motif of type I which is predominant in European (hpEurope) strains. While the type I motif was absent from North Vietnam, it was found in 8/49 (16%) of the samples from Ho Chi Minh city near the Mekong delta. Interestingly, prior to annexation by the Vietnamese in the 17^th^ century, this city was an important Khmer sea port known as Prey Nokor [4]. Thus, hpEurope strains also seem to be frequent among Vietnamese in the Mekong delta, and thus the Annamite mountain range that originates in the Tibetan and Yunnan regions of southwest China and forms Vietnam's border with Laos and Cambodia seem to have shaped an effective natural barrier for the containment of Indian influence in the Mekong basin, explaining the low prevalence of hpEurope strains elsewhere in Vietnam.

Our data are the first evidence of an important ancient genetic Indian influx this far south in Southeast Asia, except for some archaeological data. Recent excavations in peninsular Thailand have provided convincing evidence that there was a settlement there from the 3rd century BC of Indian artisans, probably of south Indian origin. Then, there was continuing Indian contact through trade and settlement throughout the period up to and including Angkor in Cambodia as well [6], [7], [8], [9]. These data are in contrast to studies on the frequencies of human mtDNA haplotypes, which despite larger sample sizes and a larger number of nearby sampling locations, showed that the genetic makeup of South-East Asian populations from Cambodia, Laos and Vietnam was largely autochthonous [10], [11], [12]. An analysis of glucose-6-phosphate dehydrogenase (G6PD) deficiency alleles in Malaysian Malays [30] identified common Southeast Asian variants (52% of the total variants) that also suggested a shared ancestral origin with the Cambodians, Laotians and Thais. Interestingly, a “Mediterranean” variant that accounts for 27% of the disease alleles among Malays [30] which is also present at low frequency in Thailand [31] and among Mon from Myanmar [32], is the most frequent variant among Indian caste groups [33]. However, this variant was not found among Khmer from Cambodia [34], and hence the “Mediterranean” G6PD deficiency allele does probably not reflect the ancient Indian genetic influx in Southeast Asia. Thus, our analysis and previous studies [16], [19], [25], [35] demonstrate that *H. pylori* genetic diversity has more discriminatory power than traditional human genetic markers in distinguishing the sources of relatively recent human migrations.

### Asian *H. pylori* in Southeast Asia

Vietnamese (Vietnam) and Khmer (Cambodia) are related languages in the sub-family Mon-Khmer of the Austro-Asiatic language family [36]. Since strains of the population hpEastAsia, subpopulation hspEAsia, were previously described as the predominant *H. pylori* in Vietnam [13], we anticipated Khmers also to carry *H. pylori* of this population which was indeed the case. Recent attention has focussed on the question of localising the Austro-Asiatic homeland, and interdisciplinary research sought evidence from linguistics, genetics, and archaeology [37], [38]. Here, we analyzed pairwise *F_ST_* values using concatened sequences of hspEAsia strains from Cambodia (32 strains), Vietnam (20 strains), Thailand (18 strains), Malaysia (25 strains), Singapore (9 strains), Japan (24 strains), Korea (10 strains), Taiwan (15 strains) and various geographic locations in China (93 strains) (Figure 2A). For comparison, we added isolates of the hspMaori population (76 strains) from native Taiwanese, Melanesians, Samoans and New Zealand Maoris, as well as isolates of the hspAmerind population (18 strains) from North and South America. The tree (Figure 2B) displayed three distinct clusters that corresponded to the three subpopulations hspEAsia (found in East Asians), hspMaori (Pacific islanders) and hspAmerind (Native Americans) in agreement with AMOVA analyses (Table S2). Within hspMaori, the tree reflects the trajectory of the Austronesian expansion that started from Taiwan and dispersed one of several hspMaori clades along with one of several subgroups of the Austronesian language family into Melanesia and Polynesia [16]. Although our data are not conclusive on the source of the Austro-Asiatic expansion, the tree topology of the subcluster hspEAsia (Figure 2C) that was supported by AMOVA analyses (Table S3) is consistent with the hypothesis that ancestors of the Austro-Asiatic people migrated from southern China into Southeast Asia, introducing hspEAsia bacteria into Vietnam and Cambodia. This language family might have been spread together with rice agriculture as part of a Neolithic human diaspora from the Yangzi and Yellow River Basins in China into Southeast Asia. The settlement of Southeast Asia has been dated from about 2000 BC [39], [40].

The origin of the hspEAsia strains from Malaysia, Thailand and Singapore is different as those were isolated from patients with Chinese origin or ancestry [13], [18] and thus reflect recent migrations within the last 200 years. Accordingly, they clustered with recent isolates from China (Figure 2B), particularly from Guangzhou and Hongkong (historically both Guangdong province), in perfect agreement with the historical origin of Malayan Chinese and Thai Chinese in China as the most of them came from Guangdong and the neighboring province Fujian. Immigrants from the same provinces made up the majority of the today's Taiwan Chinese which is also reflected in the tree.

**Figure 3:**
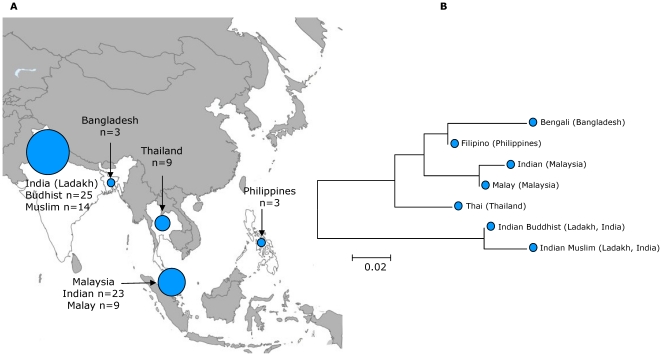
The distribution hpAsia2 haplotypes in Southeast Asia. (A) Sampling locations of hpAsia2 haplotypes in Southeast Asia. (B) A neighbor-joining tree constructed from pair-wise *F_ST_* values of hpAsia2 haplotypes indicates a common ancestral origin of hpAsia2 strains from Thailand, Bangladesh, Malaysia and the Philippines.

The remaining *H. pylori* strains isolated from Malaysia and Thailand were assigned to hpAsia2. If an ancient migration from India was the source, hpAsia2 strains would be expected to be widespread in Cambodia. However, this genetic population was absent in isolates from Khmer people. Then, we calculated pairwise *F_ST_* values between pairs of labelled populations from Thailand (9 strains), from Malaysia (32 strains), from Bangladesh (3 strains), from North India (Ladakh) (39 strains) and from the Philippines (3 strains), and generated a neighbor-joining tree (Figure 3A). As expected, isolates from Buddhists and Muslims from Ladakh in North India clustered together. However, due to substantial introgression of nucleotides from East Asian *H. pylori* [13], [19], these isolates are strongly differentiated from other hpAsia2 populations. hpAsia2 strains from Thailand, Bangladesh, Malaysia and the Philippines clustered together in the neighbor-joining tree indicating a common ancestral origin, which was supported by the AMOVA analyses (Table S4). Based on the tree topology and the absence of hpAsia2 strains in Vietnam and Cambodia (Figure 3B), we propose that two migrations introduced hpAsia2 strains into Southeast Asia, a first migration of the ancestors of the Thai people during the early second millennium AD from southern China into Thailand [3], [4], [5], and a recent migration of Indians to Malaysia (see above), carrying the bacteria into a pre-existing Malay population with low *H. pylori* carriage, in agreement with Tay et al [18].

### Strain competition and subversion, host range expansion

The absence of Western Asian lineages in human mtDNA from Southeast Asia [10], [11], [12] indicates that this ancient migration from India alone does not explain such a high frequency of hpEurope strains. Host range expansion has been described in South-America with the displacement of hspAmerind strains by hpEurope strains due to strain competition or strain subversion by transformation, integrating DNA from other strains [14], [41], [42], [43]. Inter-strain recombination which has been identified as the major driving force behind allelic diversity in *H. pylori* is critically dependent on the frequent occurrence of mixed infections, which seem to be common in developing countries [44], [45], [46]. The re-shuffling of the genetic material generates organisms that can inhabit a wide array of niches (generalist strains), and the fittest strains, e.g. of the population hpEurope, will eventually outcompete the specialist strains, e.g. of the population hspAmerind, that lack the necessary genetic diversity to efficiently colonize a wide host spectrum (specialist strains) [42].

The low prevalence of hspEAsia strains among ethnic Thai (0 out of 14 strains) [13], [47] and Malays (2 out of 15 strains) [18], despite early Chinese and Khmer influences [4], [5], [27], indicates specialist strains with a lower ability to adapt to a wide range of human hosts. In contrast, the observed host range expansion of hpEurope strains in Southeast Asia, as well as their spread among South American Amerinds and mestizos [42], indicates that these are generalist strains with a broad host spectrum. Subversion of hpEastAsia strains by transformation with DNA from hpEurope strains eventually changes those into hpEurope strains thereby further broadening the host range. The high frequency of hpAsia2 strains in Malays (9 out of 15 strains) suggests strains with a higher ability than hspEAsia strains to adapt to a wide range of human hosts and/or stronger interactions between Malays and Malaysian Indians than between Malays and Malaysian Chinese.

### *H. pylori* populations and the incidence rate of gastric cancer

Gastric carcinoma (GC), the fourth most common cancer worldwide is the second leading cause of cancer-related deaths [48]. The highest age standardized incidences (ASR) have been described in Asia but regional variations exist [49] that do not match the distribution of infection prevalence rates except for Malaysia [50]. Even if the clinical outcome of *H. pylori* infection is a complex process, the regional variations of GC incidence within Asia seem to be closely related to the distribution of the *H. pylori* genetic populations. In countries where almost all the strains are assigned to hspEasia (Japan, China, Korea, and Vietnam) [14], the incidence of GC is high (ASR 18.9 to 41.4/100 000). In contrast, incidence is low (ASR 3.5 to 5.2/100 000) in countries with a high proportion of hpAsia2 or hpEurope strains (India and Thailand) [13], [17], [18]. Cambodia that displays a mixture of hpEurope and hspEAsia strains is classified among countries with intermediate risk of GC (ASR 9.8/100 000) [49]. The genetic background might be a marker of virulence factors directly involved in clinical outcome. Further studies are needed to investigate *H. pylori* virulence factors. In the future, human mobility combined with the host range expansion of hpEurope strains may accelerate the genetic admixture of *H. pylori* populations, and thus may have a significant impact on GC incidence in Asia.

**Figure 4:**
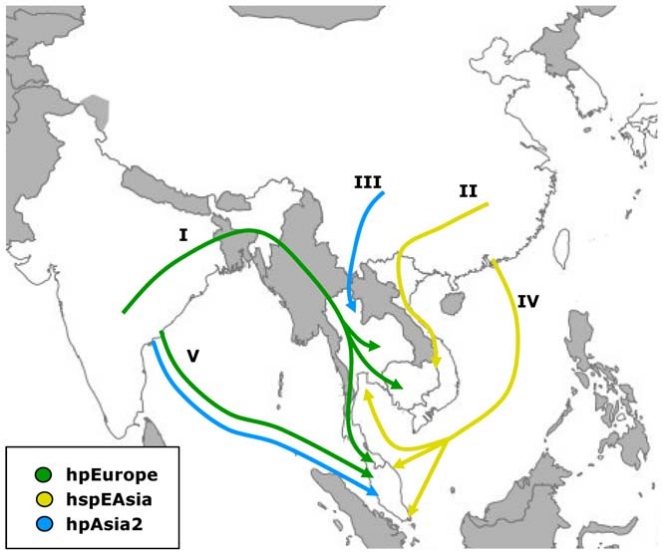
Human migrations in Southeast Asia as proposed from *H. pylori* sequences. I) An ancient migration from India distributed hpEurope bacteria in Southeast Asia. II) An ancient migration of Austro-Asiatic speakers from China carrying bacteria of the population hspEAsia. III) A migration of Tai-Kadai speakers introduced hpAsia2 bacteria in Thailand. IV) Recent migrations of Chinese from the Guangdong and Fujian provinces spread hspEAsia bacteria in Malaysia and Thailand within the last 200 years. V) Recent migrations of Indians to Malaysia brought both hpEurope and hpAsia2 bacteria to Malaysia.

In conclusion, Southeast Asia was probably free of *H. pylori* before major human migrations. These movements included (Figure 4) i) an ancient migration from India introducing hpEurope bacteria into Thailand, Cambodia and Malaysia; ii) an ancient migration of the ancestors of Austro-Asiatic people from China into Vietnam and Cambodia carrying hspEAsia bacteria; iii) an ancient migration of the ancestors of the Thai people into Thailand carrying *H. pylori* of population hpAsia2; iv) a recent migration of Chinese from the Guangdong and Fujian provinces into Southeast Asia spreading hspEasia strains; and v) a recent migration of Indians to Malaysia carrying both hpAsia2 and hpEurope bacteria.

## Supporting Information

Figure S1 **Distruct plot of the proportions of ancestral nucleotides in** ***H. pylori*** **isolates from India, Thailand, Cambodia, Vietnam and China according to the ethnic group or the religion, as determined by Structure V2.0 (linkage model).** A vertical line for each isolate indicates the estimated amount of ancestry from each ancestral population as five coloured segments. Vertical black lines separate the individuals into (sub)-populations, as determined by the no-admixture model in Structure V2.0.
(TIF)

Table S1 **AMOVA analyses for hpEurope isolates.**
(DOC)

Table S2 **AMOVA analyses for hpEastAsia isolates.**
(DOC)

Table S3 **AMOVA analyses for hpEastAsia.**
(DOC)

Table S4 **AMOVA analyses for hpAsia2.**
(DOC)
