# Supplementary figures and images for: Evolutionary History of Helicobacter pylori Sequences Reflect Past Human Migrations in Southeast Asia
Source: PLoS One. 2011 Jul 19;6(7):e22058. doi: 10.1371/journal.pone.0022058 (PMC3139604; doi:10.1371/journal.pone.0022058)

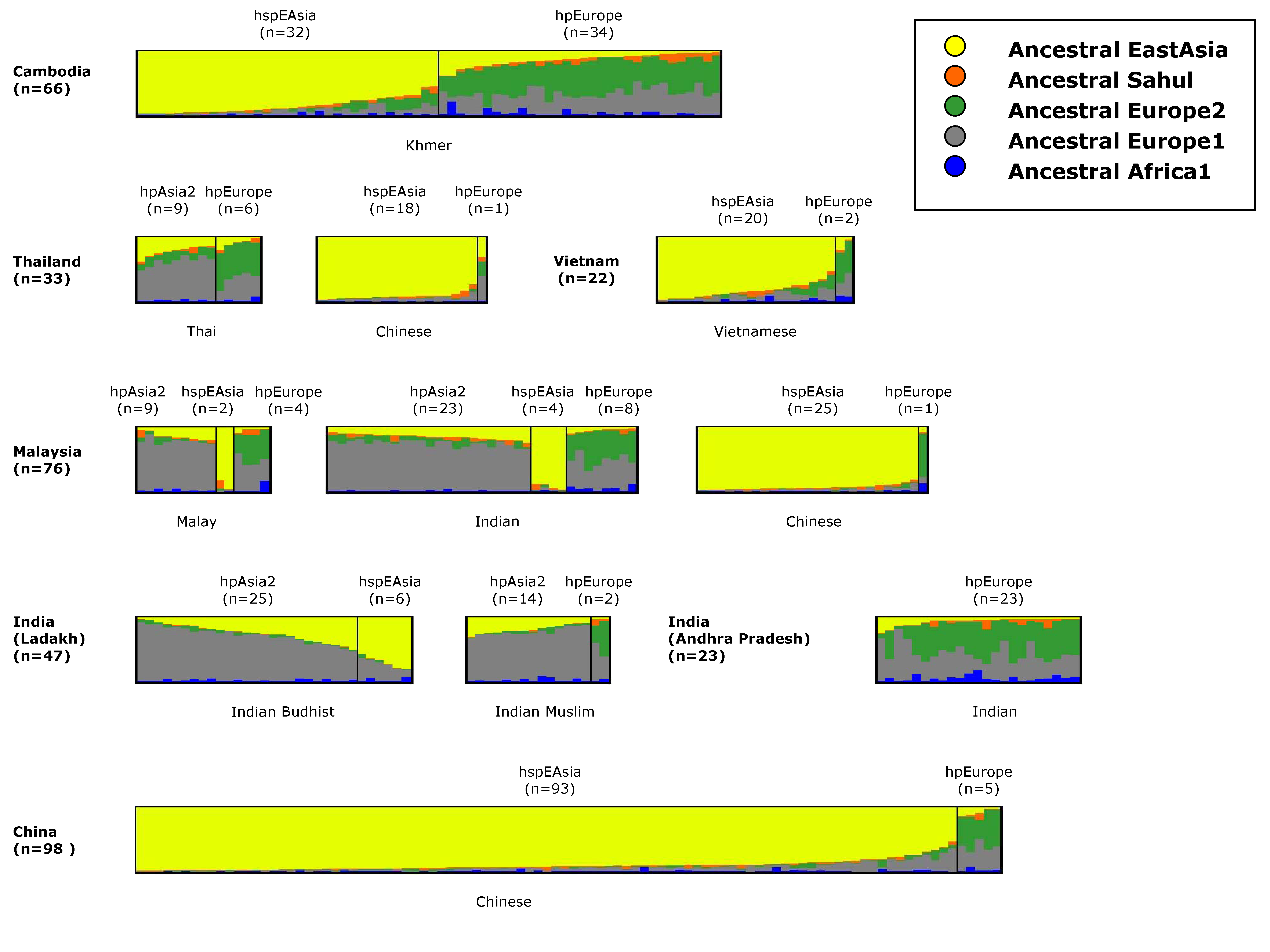

Supplement: Figure S1 — Distruct plot of the proportions of ancestral nucleotides in H. pylori isolates from India, Thailand, Cambodia, Vietnam and China according to the ethnic group or the religion, as determined by Structure V2.0 (linkage model). A vertical line for each isolate indicates the estimated amount of ancestry from each ancestral population as five coloured segments. Vertical black lines separate the individuals into (sub)-populations, as determined by the no-admixture model in Structure V2.0. (TIF) [file pone.0022058.s001.tif]
