# Supplementary material for: Evolutionary History of Helicobacter pylori Sequences Reflect Past Human Migrations in Southeast Asia
Source: PLoS One. 2011 Jul 19;6(7):e22058. doi: 10.1371/journal.pone.0022058 (PMC3139604; doi:10.1371/journal.pone.0022058)
Supplement: Table S1 — AMOVA analyses for hpEurope isolates. (DOC) [file pone.0022058.s002.doc]

Table S1. AMOVA analyses for hpEurope isolates (Figure 1B)

| **Source** | **Source assigned to group** | | | | | | |
| --- | --- | --- | --- | --- | --- | --- | --- |
| Estonia | 1 | 1 | 1 | 1 | 1 | 1 | 1 |
| Finland | 1 | 1 | 1 | 1 | 1 | 1 | 1 |
| UK | 1 | 1 | 1 | 1 | 1 | 1 | 1 |
| Germany | 1 | 1 | 1 | 1 | 1 | 1 | 1 |
| Netherlands | 1 | 1 | 1 | 1 | 1 | 1 | 1 |
| Russia | 1 | 1 | 1 | 1 | 1 | 1 | 1 |
| Kazakhstan | 1 | 1 | 1 | 1 | 1 | 1 | 1 |
| Iran | 1 | 1 | 1 | 1 | 1 | 1 | 1 |
| Turkey | 1 | 1 | 1 | 1 | 1 | 1 | 1 |
| Italy | 1 | 1 | 1 | 1 | 1 | 1 | 1 |
| Lebanon | 1 | 1 | 1 | 1 | 1 | 1 | 1 |
| Palestine | 1 | 1 | 1 | 1 | 1 | 1 | 1 |
| France | 2 | 2 | 2 | 2 | 2 | 2 | 4 |
| Spaniard | 2 | 2 | 2 | 2 | 2 | 2 | 2 |
| Basque | 2 | 2 | 2 | 2 | 2 | 2 | 2 |
| Philippines | 2 | 3 | 2 | 2 | 2 | 2 | 2 |
| Indian | 3 | 3 | 3 | 3 | 3 | 3 | 3 |
| Malaysia Indian | 3 | 3 | 3 | 3 | 3 | 3 | 3 |
| Malaysia Malay | 3 | 3 | 3 | 2 | 4 | 3 | 4 |
| Thailand | 3 | 3 | 3 | 2 | 4 | 4 | 4 |
| Cambodia | 3 | 3 | 2 | 2 | 4 | 4 | 4 |
| ***F*_ST_** | 0.04841 | 0.04701 | 0.04529 | 0.04539 | **0.04948** | 0.04923 | 0.04884 |

The highest *F*_ST_ value (in bold) indicates the most likely group assignment supporting the Neighbor-joining tree in Fig. 1.
