# Supplementary material for: Evolutionary History of Helicobacter pylori Sequences Reflect Past Human Migrations in Southeast Asia
Source: PLoS One. 2011 Jul 19;6(7):e22058. doi: 10.1371/journal.pone.0022058 (PMC3139604; doi:10.1371/journal.pone.0022058)
Supplement: Table S2 — AMOVA analyses for hpEastAsia isolates. (DOC) [file pone.0022058.s003.doc]

Table S2. AMOVA analyses for hpEastAsia isolates (Figure 2B)

| ***Source*** | **Source assigned to group number** | | | | | |
| --- | --- | --- | --- | --- | --- | --- |
| Amerindian North | 1 | 1 | 1 | 1 | 1 | 1 |
| Amerindian South | 1 | 1 | 1 | 1 | 1 | 1 |
| Taiwanese Aboriginal | 2 | 1 | 1 | 1 | 1 | 2 |
| Melanesian Malanesia | 2 | 2 | 2 | 2 | 2 | 2 |
| Polynesian Wallis | 2 | 2 | 2 | 2 | 2 | 2 |
| Polynesian Samoa | 2 | 2 | 2 | 2 | 2 | 2 |
| New Zealand Maori | 2 | 2 | 2 | 2 | 2 | 2 |
| Korean Korea | 3 | 3 | 3 | 3 | 3 | 3 |
| Japanese Hoshu | 3 | 3 | 3 | 3 | 3 | 3 |
| Japanese Hokkaido | 3 | 3 | 3 | 3 | 3 | 3 |
| Japanese Okinawa | 3 | 3 | 3 | 3 | 3 | 3 |
| Indian Ladakh | 3 | 3 | 3 | 3 | 3 | 3 |
| Chinese Beijing | 3 | 3 | 3 | 3 | 3 | 3 |
| Chinese Heilongjiang | 3 | 3 | 3 | 3 | 3 | 3 |
| Chinese Hangzhou | 3 | 3 | 3 | 3 | 3 | 3 |
| Chinese Xian | 3 | 3 | 3 | 3 | 3 | 3 |
| Chinese Yunnan | 3 | 3 | 3 | 3 | 3 | 3 |
| Chinese Chongqing | 3 | 3 | 3 | 3 | 3 | 3 |
| Chinese HongKong | 3 | 4 | 4 | 4 | 4 | 3 |
| Chinese Guangzhou | 3 | 4 | 4 | 4 | 4 | 3 |
| Chinese Taiwan | 3 | 4 | 4 | 4 | 4 | 3 |
| Chinese Thailand | 3 | 4 | 5 | 5 | 4 | 3 |
| Chinese Malaysia | 3 | 4 | 5 | 5 | 4 | 3 |
| Chinese Siangapore | 3 | 4 | 5 | 5 | 4 | 3 |
| Vietnamese Vietnam | 3 | 3 | 3 | 3 | 4 | 2 |
| Khmer Cambodia | 3 | 3 | 3 | 3 | 4 | 2 |
| ***F*_ST_** | **0.23006** | 0.17866 | 0.17514 | 0.17514 | 0.16868 | 0.16715 |
