# Supplementary material for: Evolutionary History of Helicobacter pylori Sequences Reflect Past Human Migrations in Southeast Asia
Source: PLoS One. 2011 Jul 19;6(7):e22058. doi: 10.1371/journal.pone.0022058 (PMC3139604; doi:10.1371/journal.pone.0022058)
Supplement: Table S3 — AMOVA analyses for hpEastAsia. (DOC) [file pone.0022058.s004.doc]

Table S3. AMOVA analyses for hpEastAsia (Figure 2C)

| ***Source*** | **Source assigned to group number** | | | | | |
| --- | --- | --- | --- | --- | --- | --- |
| Chinese Beijing | 1 | 1 | 1 | 1 | 1 | 1 |
| Chinese Heilongjiang | 1 | 1 | 1 | 1 | 1 | 1 |
| Chinese Hangzhou | 1 | 1 | 1 | 1 | 1 | 1 |
| Chinese Xian | 1 | 1 | 1 | 1 | 1 | 1 |
| Chinese Yunnan | 1 | 1 | 1 | 3 | 1 | 1 |
| Chinese Chongqing | 2 | 2 | 2 | 2 | 2 | 2 |
| Chinese HongKong | 2 | 2 | 2 | 2 | 2 | 2 |
| Chinese Guangzhou | 2 | 2 | 2 | 2 | 3 | 2 |
| Chinese Taiwan | 2 | 2 | 2 | 2 | 2 | 2 |
| Chinese Thailand | 2 | 2 | 2 | 2 | 2 | 3 |
| Chinese Malaysia | 2 | 2 | 2 | 2 | 2 | 3 |
| Chinese Siangapore | 2 | 2 | 2 | 2 | 2 | 3 |
| Vietnamese | 2 | 1 | 3 | 3 | 3 | 2 |
| Khmer Cambodia | 2 | 1 | 3 | 3 | 3 | 2 |
| ***F*_ST_** | **0.03597** | 0.03247 | 0.03226 | 0.03104 | 0.03011 | 0.03264 |
