# Supplementary material for: Evolutionary History of Helicobacter pylori Sequences Reflect Past Human Migrations in Southeast Asia
Source: PLoS One. 2011 Jul 19;6(7):e22058. doi: 10.1371/journal.pone.0022058 (PMC3139604; doi:10.1371/journal.pone.0022058)
Supplement: Table S4 — AMOVA analyses for hpAsia2. (DOC) [file pone.0022058.s005.doc]

Table S4. AMOVA analyses for hpAsia2 (Figure 3B)

| **Source** | **Source assigned to group number** | | | | | |
| --- | --- | --- | --- | --- | --- | --- |
| Indian Buddhist, Ladakh | 1 | 1 | 2 | 1 | 1 | 1 |
| Indian Muslim, Ladakh | 1 | 1 | 1 | 1 | 1 | 1 |
| Thai Thailand | 2 | 2 | 2 | 2 | 2 | 2 |
| Malay Malaysia | 2 | 2 | 2 | 2 | 2 | 3 |
| Indian Malaysia | 2 | 2 | 1 | 1 | 1 | 3 |
| Philippino Phillipines | 2 | 3 | 2 | 2 | 3 | 3 |
| Bengali Bangladesh | 2 | 3 | 2 | 2 | 3 | 3 |
| ***F*_ST_** | **0.18332** | 0.17059 | 0.12167 | 0.14854 | 0.14345 | 0.17073 |
